# Nationwide survey of Indian cardiac surgeons on the management of acute type A aortic dissection

**DOI:** 10.1016/j.sipas.2025.100316

**Published:** 2025-10-17

**Authors:** Mohammed Idhrees, Nimrat Grewal, Mohammed Ayyub, Jasima Nilofer, Bashi Velayudhan

**Affiliations:** a Institute of Cardiac and Aortic Disorders (ICAD), SRM Institutes for Medical Science (SIMS Hospital), Chennai, India; b Clinical Research, Institute of Cardiac and Aortic Disorders (ICAD), SRM Institutes for Medical Science (SIMS Hospital), Chennai, India; c Department of Cardiothoracic Surgery, Amsterdam University Medical Center, Amsterdam, The Netherlands; d Department of Cardiothoracic Surgery, Leiden University Medical Center, Amsterdam, The Netherlands; e Section of Cardiac Surgery, Yale University School of Medicine, New Haven, CT, USA; f Department of Respiratory Medicine, Sri Ramachandra Institute of Higher Education and Research, Chennai, India; g Department of Pathology, Psp Medical College Hospital and Research Institute, Chennai, India

**Keywords:** Aortic dissection, Aortic surgery, Aortic arch, Malperfusion, cerebral perfusion, hypothermic circulatory arrest, survey

## Abstract

**Objective:**

Acute Type A aortic dissection (ATAAD) is associated with high morbidity and mortality, and management strategies vary widely among surgeons. This study aimes to evaluate practice patterns and decision-making among Indian cardiac surgeons regarding ATAAD, with focus on differences related to surgical experience and institutional case volume.

**Methods:**

A 23-item electronic questionnaire covering preoperative, intraoperative and postoperative management of ATAAD was distributed to all members of the Indian Association of Cardiovascular-Thoracic Surgeons. Ninety-three responses were analyzed and compared according to surgeon experience (<10, 10–20, >20 years) and institutional aortic surgery volume (high vs low).

**Results:**

Over one-quarter of surgeons (26.9 %) declined to operate on patients >70 years old, a practice more frequent among surgeons with <20 years of experience (32% vs 5.56 % p=0.011). Active cardiopulmonary resuscitation (56 %), and preoperative stroke (52.7%) were the most common reasons to withhold surgery, whereas senior surgeons (>20 years) were more likely to operate despite malperfusion or CPR (38.8 % vs 13.3 %, p=0.005).

Dual arterial cannulation was preferred by 62.4% of surgeons, with a shift toward single site cannulation with increasing experience (p=0.008). The distal anastomosis was performed using on-clamp technique by 26.8 % of respondents, more frequently among low-volume aortic surgeons (35.1 % vs 13.8%, p=0.012).

**Conclusion:**

Management of ATAAD in India shows substantial variation, strongly influenced by surgeon experience and aortic surgery volume. Differences are particularly evident in patients selection, cannulation strategy and distal repair techniques. These findings highlight the need for structured referral systems and the potential benefit of developing high-volume ‘aortic centres’ in India.

## Introduction

Acute type A aortic dissection (ATAAD) is among the most dramatic and life-threatening cardiovascular emergencies, with an untreated mortality of up to 90 % [1,2]. Prompt diagnosis and urgent surgical repair remain the gold standard, offering the only chance to prevent fatal complications such as rupture, tamponade, or irreversible malperfusion [3]. Despite advances in imaging, cardiopulmonary bypass technology, and perioperatieve care, overall outcome remain modest, reflecting both the aggressive natural history of the disease and the complexity of surgical repair [2].

Management of ATAAD is heterogeneous across the globe, influenced by differences in hospital resources, referral patterns and surgeon experience [4]. Critical decisions include whether to proceed with surgery in elderly of hemodynamically unstable patients, the choice of cannulation strategy, cerebral protection methods, and the extent of aortic resection. Postoperative management, including the use of anticoagulation and imaging surveillance, also varies considerably between centres.

India faces unique challenges in the care of ATAAD. The country’s vast geography and heterogeneous healthcare infrastructure contribute to delayed diagnosis and transfer, while financial limitations may further affect timely intervention [5]. Moreover, the lack of centralized “aortic centres,” as seen in some Western countries, results in both high- and low-volume centres managing these critically ill patients which could potentially influencing outcomes.

To better understand current practice of ATAAD management in India, we conducted a nationwide survey of Indian cardiothoracic surgeons. The survey focused on how preoperative selection, intraoperative strategies, and postoperative care vary according to surgeon experience and institutional aortic case volume. Identifying these patterns can help inform national strategies for centralization, standardization, and training, ultimately aiming to improve ATAAD outcomes in India.

## Methods

### Study aim

The primary aim of this study was to assess contemporary practice patterns among cardiothoracic surgeons in India with regarding the management of to ATAAD. We analysed variations in clinical preferences according to surgeons’ experience, overall cardiac surgical volume of the institution, and aortic surgery volume.

### Study design and data collection

We conducted an observational, cross-sectional survey of cardiothoracic surgeons practicing in India. An 23-item questionnaire was created using Google Forms, covering preoperatieve, intraoperative, and postoperative aspects of ATAAD management. The survey link was distributed by email to all the registered members of the Indian Association of Cardiovascular-Thoracic Surgeons starting from May 13th, 2021. To maximize participation, several reminders were sent by the authors, and the survey was also shared via WhatsApp. Participation was voluntary and anonymized.

### Study population

The target population included all cardiothoracic surgeons actively practising in India.

Inclusion criteria: All fully completed questionnaires were included for analysis.

Exclusion criteria: Responses from (i) Indian cardiothoracic surgeons practicing abroad, and (ii) residents, fellows, or research assistants without independent surgical practice were excluded.

### Statistical analysis

Comparisons between different groups were performed using Pearson’s chi-square test or Fisher exact test, as appropriate. A p-value <0.05 was considered statistically significant. The analysis was carried out using SPSS v.22.0

### Definitions

• High-volume surgeon: ≥200 total cardiac surgeries per year.
• High-volume aortic surgeon: ≥10 % of total surgical practice dedicated to aortic surgery.
• Experience categories:
  ○ Young: <10 years
  ○ Mid-career: 10–20 years
  ○ Senior: >20 years

### Ethical statement

This study involved a voluntary, anonymized survey of cardiovascular and thoracic surgeons of India, without patient participation. No human or animal experiment were conducted. The protocol was approved by the institutional ethics committee.

## Results

### Surgeon demographics

**Fig 1:**
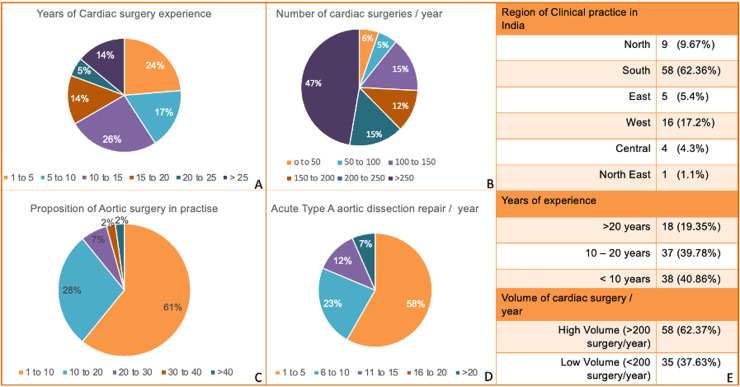
Demographic characteristics and surgical practice patterns of the participating Indian cardiothoracic surgeons. Panel A shows the distribution of years of cardiac surgery experience, with the largest proportion (26 %) having 10–15 years in practice, followed by 24 % with 1–5 years and 17 % with 20–25 years of experience. Panel B illustrates the annual number of cardiac surgeries performed by respondents, with nearly half (47 %) performing >250 cases per year. Panel C presents the proportion of aortic surgery in the surgeons’ total practice, revealing that the majority (61 %) dedicate only 1–10 % of their caseload to aortic procedures. Panel D displays the annual number of acute type A aortic dissection (ATAAD) repairs, with 58 % of surgeons performing only 1–5 such operations per year. Panel E summarizes the regional distribution of clinical practice, years of experience, and surgical volume. Most respondents are based in southern India (62.36 %), with 40.86 % having less than 10 years of experience. High-volume surgeons, defined as performing more than 200 cardiac surgeries per year, comprised 62.37 % of the cohort.

A total of 93 cardiothoracic surgeons form across India responded to the survey (Fig 1). Of these, 58 (62.37 %) were high volume surgeons, while 35 (37.63 %) of the respondents were low volume (Fig 1). The distribution of surgical experience was:

• Young (<10 years): 38 (40.9 %)
• Mid-career (10–20 years): 37 (39.8 %)
• Senior (>20 years): 18 (19.4 %)

Nearly half of the surgeons (47 %) reported performing >250 cardiac surgeries annually, whereas only 6 % reported <50 cases. The majority (61 %) indicated that aortic surgery comprised just 1–10 % of their practice, with very few (>2 %) performing >40 % aortic cases. Annual ATAAD repair volume was low overall, with 58 % performing only 1–5 such operations, 23 % performing 6–10, and only 7 % reporting >20 cases per year. Geographically, most surgeons were located in South India (62 %), followed by West (17 %), North (10 %), East (5 %), Central (4 %), and North-East (1 %) (Fig 1).

### Preoperative considerations

Only 5 surgeons (5.4 %) reported they would proceed with surgery without a computed tomography angiography, if echocardiography demonstrated a clear dissection flap and the patient presented with classical clinical features. All five were low-volume surgeons, with aortic cases representing <10 % of their total cardiac surgery practice.

Late presentation to hospital was reported as the leading cause of preoperative mortality by 70 respondents (75 %). After admission, 18 surgeons (19.4 %) reported that >20 % of patients die before surgery. This was significantly less common among senior surgeons compared to those with <20 years’ experience (5.6 % vs 24 %, p=0.04).

**Fig 2:**
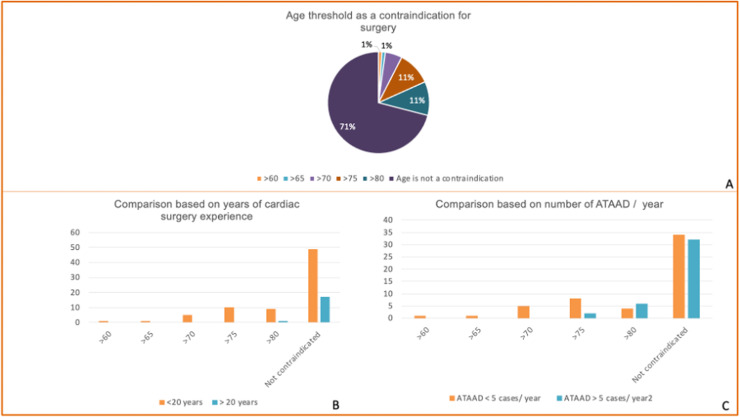
Surgeons’ views on age as a contraindication for surgery in acute type A aortic dissection (ATAAD).(A) Distribution of respondents’ self-reported upper age limit beyond which they would consider age a contraindication to surgery. Most surgeons (71 %) reported no age-based contraindication, whereas thresholds of >60, >65, >70, >75, and >80 years were reported by smaller proportions. (B) Comparison of age thresholds according to surgeons’ experience (<20 years vs ≥20 years). Surgeons with ≥20 years of experience were less likely to use age as a surgical contraindication. (C) Comparison of age thresholds according to annual ATAAD case volume (<5 vs ≥5 cases/year). Surgeons with higher ATAAD volumes tended to operate irrespective of patient age more often than their lower-volume counterparts.

Age alone was not considered a contraindication by 66 respondents (71 %). However, 25 surgeons (26.9 %) declined surgery for patients ≥70 years, a practice significantly more frequent among surgeons with <20 years’ experience (32 % vs 5.6 %, p=0.011). In centers performing >6 ATAAD cases annually, the refusal rate for patients ≥70 years was 20 % versus 32 % in centers performing ≤5 cases (p=0.20) (Fig 2). Most surgeons therefore did not consider age per se prohibitive, but younger and lower-volume surgeons were more conservative, whereas senior and higher-volume surgeons tended to operate irrespective of age.

The most commonly cited absolute contraindications to surgery were:

• Active cardiopulmonary resuscitation (CPR) – 56 %
• Stroke – 52.7 %
• Mesenteric ischemia – 41.8 %

**Fig 3:**
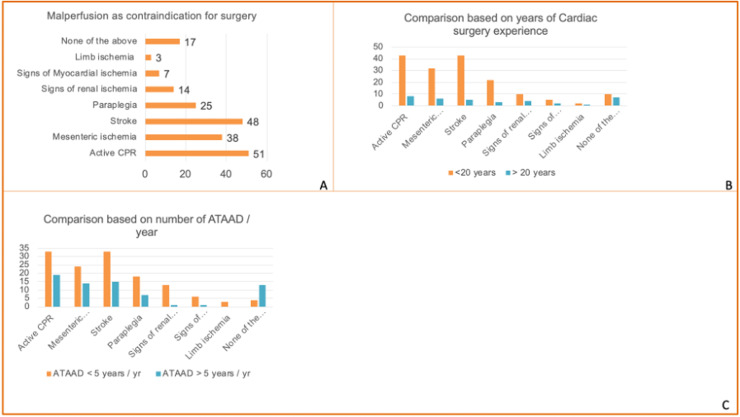
Contraindications to operative management of acute Type A aortic dissection (ATAAD). Panel A shows the distribution of malperfusion syndromes and active cardiopulmonary resuscitation (CPR) considered as contraindications for surgery. Stroke (48 %), mesenteric ischemia (38 %), and active CPR (51 %) were the most frequently reported reasons to withhold surgery, whereas limb ischemia (3 %) and myocardial ischemia (7 %) were rarely cited; 17 % of respondents reported no malperfusion as a contraindication. Panel B compares these contraindications between surgeons with <20 years and ≥20 years of cardiac surgery experience, showing that senior surgeons were less likely to view malperfusion as prohibitive. Panel C compares the same contraindications based on institutional ATAAD surgical volume (<5 vs. ≥5 cases/year), indicating that higher-volume surgeons tended to operate on a broader spectrum of malperfusion presentations.

Seventeen surgeons (18.3 %) reported operating ATAAD patients irrespective of malperfusion or CPR. Senior surgeons were significantly more likely to adopt this approach (38.9 % vs 13.3 %, p=0.005). Stroke, mesenteric ischemia, and CPR were therefore the predominant reasons to withhold surgery, whereas myocardial ischemia (7 %), renal ischemia (14 %), and limb ischemia (3 %) were seldom reported as absolute contraindications. High-volume surgeons in particular rarely excluded patients based on malperfusion syndromes, unlike their low-volume counterparts (myocardial 11.3 %, renal 22.6 %, limb 5.7 %) (Fig 3).

### Intraoperative management

#### Cannulation strategy

Dual arterial cannulation was preferred by 58 surgeons (62.36 %), most commonly prefer dual femoral plus axillary artery (n=47, 50.5 %). Single-site cannulation was more frequent among senior surgeons than those with <20 years’ experience (66.67 % vs 30.67 %, p=0.002. Axillary artery was used as the sole site for perfusion by 15.1 % of surgeons. Femoral-only cannulation was reported by 14 %, and femoral plus ascending aorta by 12 %, while only 2 % used other single-site cannulation strategies. The use of dual sites was more common among younger surgeons, whereas senior surgeons favoured a single site, reflecting an experience-related practice shift.

**Fig 4:**
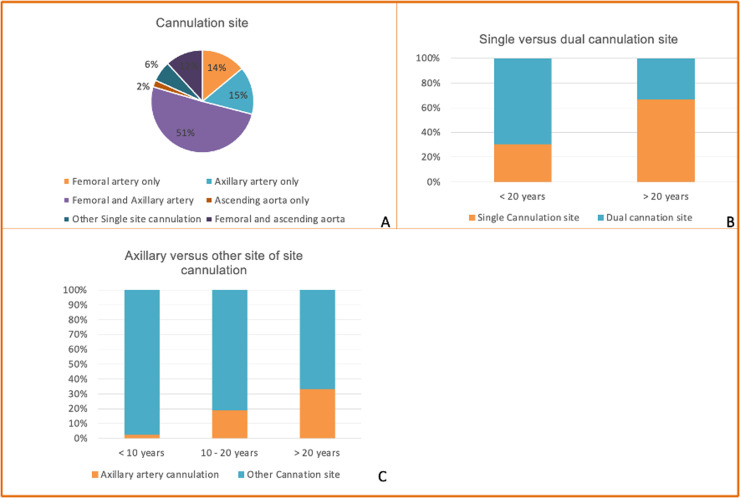
Preferred arterial cannulation strategies among respondents. (A) Distribution of preferred cannulation sites, showing that the majority of surgeons favour femoral + axillary artery cannulation, followed by single-site axillary or femoral cannulation. (B) Comparison of single versus dual-site cannulation according to surgical experience (<20 years vs ≥20 years), illustrating a higher use of dual cannulation among less experienced surgeons. (C) Use of axillary artery cannulation compared with other sites, stratified by years of experience, indicating increased adoption of axillary cannulation with greater experience.

Pre-sternotomy arterial and venous cannulation was performed by 38 surgeons (40,9 %), with another n=15 (16,1 %) cannulation before pericardial opening. Low-volume centers were more likely to perform pre-sternotomy cannulation than high-volume centers (51.4 % vs 34.5 %, p=0.05) (Fig 4).

#### Cerebral protection

Deep hypothermia (<20 °C nasopharyngeal temperature) is preferred by more than half of the participating surgeons (n=51, 54.89 %), with no significant difference by surgeon experience or volume. Antegrade cerebral perfusion (ACP), unilateral or bilateral, was the preferred neuroprotection method for 63 respondents (68 %).

#### Extent of resection

Most surgeons (n=58, 62,4 %) replaced the aortic root if aortic insufficiency was moderate or severe. Conversely, 20. % replaced only the ascending aorta with or without valve replacement even with moderate aortic insufficiency, apractice more common among surgeons with <20 years’ experience (24 % vs 5.56 %. P = 0.04)

**Fig 5:**
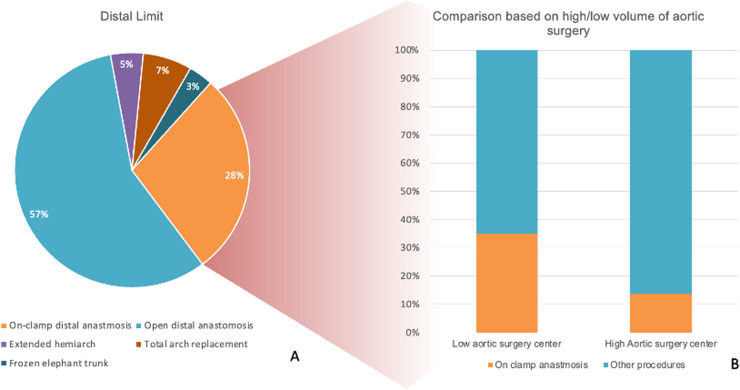
Distal limit of aortic repair in acute type A aortic dissection. (A) Distribution of preferred distal repair strategies among respondents, showing that the majority opted for open distal anastomosis (57 %), followed by on-clamp distal anastomosis (28 %), extended hemiarch (5 %), total arch replacement (7 %), and frozen elephant trunk (3 %). (B) Comparison between high- and low-volume aortic surgery centers, demonstrating that on-clamp distal anastomosis was more frequently used in low-volume centers, whereas high-volume centers more often performed alternative techniques such as open distal anastomosis or arch replacement.

For distal repair, 54,8 % preferred open distal anastomosis. The on-clamp distal anastomosis technique was used by 26.8 %, significantly more frequent in low-volume centers (35.1 % vs 13.9 %, p=0.012). Extended hemiarch repair was selected by 5 %, total arch replacement by 7 %, and frozen elephant trunk by 3 %, reflecting a minority but important subset of practice (Fig 5).

#### Malperfusion

  ð Limb ischemia: 82.8 % (n=77) performed central repair first, with delayed peripheral intervention if needed.
  ð Renal ischemia: 78 % adopted central repair first, with delayed renal intervention. All senior surgeons followed this approach.

### Post op management

**Fig 6:**
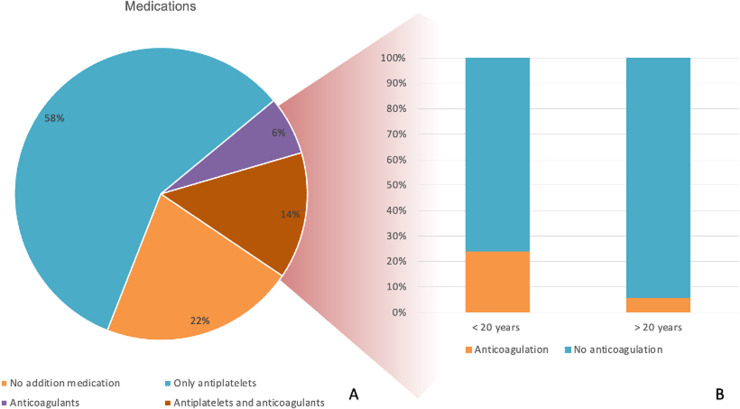
Postoperative medication strategies following aortic repair without concomitant valve replacement or coronary artery bypass grafting. (A) Distribution of prescribed medications, including no additional medication, antiplatelets only, anticoagulants only, and combined antiplatelet + anticoagulant therapy. (B) Comparison of anticoagulation use between surgeons with < 20 years and > 20 years of clinical experience.

Fifty-eight percent (n= 54, 58.06 %) of participating surgeons prefer to place patients at least on antiplatelet medications even if there is no prosthetic valve or associated coronary artery bypass grafting. After reconstructing the aorta with a dacron graft, nineteen surgeons (20.43 %) prefer anticoagulation even when there is no prosthetic valve. Surgeons with more than 20 years of experience avoid anticoagulation if there is not prosthetic valve implanted (5.56 % vs 24 %, p=0.04) (Fig 6). When stratified by regimen, 22 % reported prescribing no additional medication, 58 % prescribed antiplatelets alone, 6 % anticoagulation alone, and 14 % a combination of both.

Less than two-third of the surgeons (63 %) indicate that they would routinely obtain baseline aortic imaging following repair of ATAAD. Of the surgeons who obtain imaging, the first baseline imaging is performed at the time of discharge by 28 %, while 30 % will obtain imaging at 3-month review.

### Postoperative management

A majority (58.1 %, n=54) prescribed antiplatelet therapy postoperatively even without prosthetic valve implantation or CABG.

Anticoagulation without a prosthetic valve was preferred by 20.4 % (n=19), primarily among surgeons with <20 years’ experience (24 % vs 5.6 %, p=0.04) (Fig 6).

Only 63 % routinely obtained baseline postoperative aortic imaging. Of these, 28 % performed imaging at discharge, and 30 % at 3 months.

## Discussion

This nationwide survey provides insight into the current management of ATAAD among Indian cardiac surgeons. While overall strategies show similarities across the country, our findings demonstrate that surgeon experience and institutional aortic volume significantly influene key aspects of management, including cannulation strategy, the treatment of malperfusion, proximal and distal repair extent, and cerebral protection startegies.

Our findings can be correlated with the recently published EACTS/STS guidelines on acute and chronic aortic syndromes, which emphasize early referral to high-volume centers, preference for open distal anastomosis, and structured imaging follow-up after repair [3]. While Indian surgeons report many similar practices, the survey also highlights important gaps in guideline adherence, particularly regarding postoperative surveillance and anticoagulation strategies.

A notable observation is that approximately one-fifth of surgeons report that >20 % of ATAAD patients die after hospital admission, but before surgery, with delayed presentation as the leading cause. This challenge is likely related to the unique context of India compared to the Western world (3).

(i) *Transport and geography:* India spans 1997 miles (north-south) and 1822 miles (east-west) with diverse terrain including hills, valleys, plateaus, deserts and rivers, making road transport slow and often poorly tolerated for critically ill patients. Air ambulance services exist mainly in major cities and have limited overall impact.
(ii) *Financial burden:* Healthcare is not fully government-funded. Patients without prior insurance often face significant “out-of-pocket” costs and income loss during hospitalization, leading to delayed consent and treatment.
(iii) *Diagnostic Centres:* Many smaller towns lack immediate CT imaging to confirm, and iths highly variable presentation increases the risk of misdiagnosis before referral to tertiary centres.

Surgeons with <20 years of experience were more likely to decline surgery in patients over 70 years of age, even though the Indian population ≥65 years is only 6.18 % (2018, World Bank), While perioperative risk is higher in septuagenarian and octogenarians, it remains lower than the natural history of untreated ATAAD, and surgery can yield acceptable outcomes if hemodynamic stability is present [[6], [7], [8]]. Tailoring surgical aggressiveness may improve outcomes in this group [9].

Malperfusion syndrome are strongly associated with adverse outcomes. Cerebral injury has been considered a relative contraindication to emergency surgery, with some authors suggesting delayed surgical intervention [10,11]. However data from the International Registry of Acute Aortic Dissection (IRAD) show dismal outcomes with medical therapy alone, 100 % mortality in patients with coma and 76.2 % in those with cerebrovascular accidents, while surgery confers a 50 % survival advantage [12]. In our survey, more than half of surgeons would not operate on patients with preoperative stroke (52.7 %). Senior surgeons and high-volume operators were more likely to operate in patients with ongoing CPR or any malperfusion (38.88 % vs 13.33 %, p=0.04), likely reflecting their accumulated expertise, high-volume centre infrastructure, and broader team support.

Cerebral protection remains a critical aspect of ATAAD repair. Over 50 % of surgeons prefer deep hypothermic circulatory arrest (DHCA), and nearly 70 % use antegrade cerebral perfusion (ACP), while 14 % still use isolated DHCA. Expert consensus classifies hypothermia for aortic surgery as profound (<14 °C), deep (14.1–20 °C), moderate (20.1–28 °C), and mild (28.1–34 °C) [13]. Although hypothermia is the cornerstone of neuroprotection, international trends favor moderate hypothermia with ACP to reduce bypass duration and coagulopathy [[14], [15], [16]]. Damberg et al. recently reported a 2 % stroke rate with isolated DHCA in 613 aortic operations, noting that DHCA duration >50 min may necessitate cerebral perfusion [17]. Our data reflect current Indian practice but underscore the need for further studies to evaluate optimal neuroprotection strategies.

Similarly, postoperative imaging surveillance remains an area for improvement. International guidelines recommend baseline CT or MR angiography before discharge and regular follow-up thereafter, whereas only 63 % of Indian surgeons in our survey reported obtaining baseline imaging, reflecting a key opportunity for standardization.

More than one-quarter of our surgeons prefer the distal anastomosis on an aortic cross-clamp - ‘clamp-on technique’- over open distal anastomosis, especially in low-volume centers. This method is technically simpler, faster, and avoids deep hypothermia, but it cannot treat secondary arch tears (present in 20–30 % of ATAAD) [[18], [19], [20]], may risk cross-clamp injury, and is associated with persistent false lumen [21,22]. Open distal anastomosis (ODA) enables inspection for additional tears, complete intrapericardial resection, and better remodeling [23,24]. While Geirsson et al [25] reported higher cerebrovascular complications with ODA, they still recommend ODA as the primary strategy for ATAAD. Conversely, Malvindi et al [24] observed improved neurologic outcomes and favorable remodeling with ODA plus cerebral perfusion. These findings reinforce ODA with hypothermic circulatory arrest as the preferred approach in most modern series.

Our study also demonstrated clear differences between high- and low-volume aortic centers, and between surgeons of varying experience. Postoperative anticoagulation strategies also varied considerably, with nearly one-fifth of surgeons prescribing anticoagulation even in the absence of prosthetic material. Evidence and consensus guidelines suggest that anticoagulation is not routinely required after isolated graft replacement unless mechanical prostheses or specific thromboembolic risk factors are present. This discrepancy underscores the importance of developing national recommendations tailored to the Indian context. We defined “high-volume” aortic surgeon as having ≥10 % aortic cases in their practice, reflecting the real-world Indian setting. In contrast to the United States, where the aortic [26], India lacks centralized aortic care. As shown in international series higher surgeon and center volumes correlate with lower mortality: <3 ATAAD cases/ year yields 27.4 % mortality vs 16.4 % in centres with >13 dissection/ year [27]. And UK data show similar benefits for surgeons preforming ≥4 dissection repairs/year (26).

We defined a “high-volume” aortic surgeon as having ≥10 % aortic cases in their practice, reflecting the real-world Indian setting. In contrast to the United States, where the “aortic supercenter” model has improved outcomes (24), India lacks centralized aortic care. As shown in international series, higher surgeon and center volumes correlate with lower mortality: <3 ATAAD cases/year yields 27.4 % mortality vs 16.4 % in centers with >13/year (25), and UK data show similar benefits for surgeons performing ≥4 repairs/year [28].

## Limitations

This survey relied on voluntary, self-reported responses, which may introduce selection and reporting bias. The questionnaire was not externally validated, and responses could not be cross-verified against actual surgical practice. Nonetheless, it provides the first nationwide overview of ATAAD management in India and highlight meaningful practice variations.

## Conclusion

Our survey reveals substantial heterogeneity in the management of ATAAD among Indian cardiac surgeons. Nearly one-fifth of patients die before surgery, and high-risk cases, such as eldery patients, malperfusion, or those requiring CPR, are more like to be manged surgically by senior or high-volume surgeons. These results underscore the need for standardized national protocols and the establishment of dedicated aortic supercenters to optimize outcomes in this time-critical and complex disease.

## Funding

No funding was received for this study.

## CRediT authorship contribution statement

**Mohammed Idhrees:** Writing – review & editing, Writing – original draft, Visualization, Methodology, Investigation, Formal analysis, Data curation, Conceptualization. **Nimrat Grewal:** Writing – review & editing, Visualization, Validation, Methodology. **Mohammed Ayyub:** Writing – review & editing. **Jasima Nilofer:** Writing – review & editing. **Bashi Velayudhan:** Writing – review & editing.

## Declaration of competing interest

The authors declare that they have no known competing financial interests or personal relationships that could have appeared to influence the work reported in this paper.

## Appendix Supplementary materials

Image, application 1
