# Supplementary material for: Nationwide survey of Indian cardiac surgeons on the management of acute type A aortic dissection
Source: Surg Pract Sci. 2025 Oct 17;23:100316. doi: 10.1016/j.sipas.2025.100316 (PMC12596210; doi:10.1016/j.sipas.2025.100316)
Supplement: Supplementary file 1 [file mmc1.docx]

Acute Type A Aortic dissection

# How many years have you been in practice as a cardiac surgeon?

## Mark only one oval.

1-5 years

5–10 years

10–15 years

15–20 years

20–25 years

>25 years

# Which part of the country do you practice?

## Mark only one oval.

North Zone ( Chandigarh, Delhi, Haryana, Himachal Pradesh, Jammu and Kashmir, Ladakh, Punjab, and Rajasthan)

East Zone (Bihar, Jharkhand, Odisha, and West Bengal)

West Zone (Dadra and Nagar Haveli, Daman and Diu, Goa, Gujarat, and Maharashtra)

South Zone (Andhra Pradesh, Karnataka, Kerala, Puducherry, Tamil Nadu, Telangana, Andaman and Nicobar Islands and Lakshadweep.

Central Zone (Chhattisgarh, Madhya Pradesh, Uttarakhand and Uttar Pradesh)

North East Zone ( Assam, Arunachal Pradesh, Manipur, Meghalaya, Mizoram, Nagaland and Tripura)

# How many cardiac cases do you perform per year?

## Mark only one oval.

0-50

50 - 100

100 - 150

150 - 200

200 - 250

>250

# What is the proportion of aortic surgery in your current practice?

## Mark only one oval.

1 - 10%

10 - 20%

20 - 30%

30 - 40%

>40%

# How many Acute type A aortic dissection patients you encounter each year?

## Mark only one oval.

A: 1-5

6-10

11-15

15

# How many Acute type A aortic dissection patients are operated at your center (without referring to tertiary center) each year?

## Mark only one oval.

1-5

6-10

11-15

15-20

>20

# What percentage of patient die before getting operated?

## Mark only one oval.

<5%

5 - 15%

15 - 20%

20 - 25%

25 - 30%

35 - 40%

# The most common reason for death prior to surgery is...........

## Mark only one oval.

Patient presented late to hospital

Patient not willing to go for further treatment Lack of expertise in the hospital

Difficulty in transporting to higher center

# In the presence of positive signs and symptoms of acute aortic dissection and a dissecting ﬂap in echocardiogram, Will you still perform CT aortogram before surgery?

## Mark only one oval.

Yes No

# Beyond which age you will not operate for patients with uncomplicated acute type A aortic dissection?

## Mark only one oval.

>60

>65

>70

>75

>80

Age alone is not a contraindication for surgery

# Which of the following factor(s) would prevent you from operating the patient with acute type A aortic dissection? [more than 1 choice permitted]

*Tick all that apply.*

Signs of Myocardial ischemia Signs of renal ischemia Mesenteric ischemia

Stroke Paraplegia Limb ischemia Active CPR

None of the above

# What is the preferred site of cannulation for repair of acute type A dissection in your practice?

## Mark only one oval.

Femoral artery only Axillary artery only Innominate artery Only Ascending aorta only Carotid artery only Femoral and Axillary artery

Femoral and ascending aorta

Other:

# What is your strategy for cannulation in stable patients?

## Mark only one oval.

Before sternotomy

Sternotomy and cannulation done simultaneously by 2 different teams Before Opening the pericardium

After opening the pericardium

# During repair of an acute type A dissection, the target lowest nasopharyngeal temperature is.

## Mark only one oval.

<18 degree Celsius

18 to 20 degree celcius

21 to 24 degree Celsius

25 to 28 degree Celsius

28 to 32 degree Celcius More than 32 degree Celcius

# Ωuring DHCA our / my primary method of cerebral protection is

## Mark only one oval.

Distal anastmosis done on aortic cross clamp Unilateral antergrade cerebral perfusion Bilateral antergrade cerebral perfusion

Retrograde cerebral perfusion

No perfusion to brain. Just cooling and anesthetic management

# Patient with acute Type A aortic dissection with more than moderate aortic regurgitation - the surgery I /we preform is. ?

## Mark only one oval.

Ascending aorta replcaement

Ascending aorta replacement with aortic valve replacement Ascending aorta replacement with aortic valve repair Bentall's Procedure

Aortic valve sparing root replacement - David's procedure

# Regarding the distal extent of aortic resection, the minimum operation that I /we perform (in addition of ascending aortic repair) is:

## Mark only one oval.

On-clamp distal anastmosis Hemiarch (open distal anastomosis) Extended hemiarch

Total arch replacement Frozen elephant trunk

# During operative repair for acute type A dissection, I use hemostatic agents

## Mark only one oval.

Never Sometimes Most of the time Always

# In patients with acute type A aortic dissections with acute renal dysfunction, my

/ our strategy is

## Mark only one oval.

Central aortic repair and if required address kidney later Endovascular stenting of renal artery followed by central aortic repair Dialysis for 4 weeks and then perform central aortic repair

# In patients with acute type A aortic dissections with limb ischemia, my / our strategy is

## Mark only one oval.

Endo vascular stent graft to thoracic / abdominal aorta and central repair at later stage

Central aortic repair and if required address limb ischemia later

# After repair of type A aortic dissection, if the patient does not have a concomitant valve, CABG or endograft, what is the medication the patient is placed on?

## Mark only one oval.

No addition medication Only antiplatelets anticoagulants

Antiplatelets and anticoagulants

# Do you routinely obtain baseline aortic imaging following repair of acute aortic dissection?

## Mark only one oval.

Yes No

# If you obtain baseline aortic imaging following repair of Type A aortic dissection, when do you obtain the ﬁrst study?

## Mark only one oval.

In hospital prior to discharge. First review - 3 months

3 – 6 months

7 – 12 months

> 1 year

Only when the patients complaints of symptoms
